# Predictors of presenteeism, absenteeism and job loss in patients commencing methotrexate or biologic therapy for rheumatoid arthritis

**DOI:** 10.1093/rheumatology/keaa027

**Published:** 2020-02-25

**Authors:** James M Gwinnutt, Sarah Leggett, Mark Lunt, Anne Barton, Kimme L Hyrich, Karen Walker-Bone, Suzanne M M Verstappen, RAMS and BRAGGSS co-investigators

**Affiliations:** k1 Centre for Epidemiology Versus Arthritis, Centre for Musculoskeletal Research, Faculty of Biology, Medicine and Health, University of Manchester, Manchester Academic Health Science Centre, Manchester; k2 Centre for Genetics and Genomics Versus Arthritis, Centre for Musculoskeletal Research, Faculty of Biology, Medicine and Health, University of Manchester, Manchester Academic Health Science Centre, Manchester; k3 NIHR Manchester Biomedical Research Centre, Manchester University NHS Foundation Trust, Manchester Academic Health Science Centre, Manchester; k4 MRC Versus Arthritis Centre for Musculoskeletal Health and Work, University of Southampton, Southampton, UK

**Keywords:** rheumatoid arthritis, work, work disability, presenteeism, absenteeism, disability

## Abstract

**Objectives:**

Work is an important health outcome. This study aimed to identify predictors of work loss, absenteeism and presenteeism over 1 year in RA patients commencing treatment with MTX or biologics.

**Methods:**

Patients aged 18–65 years in full/part-time employment from two UK prospective cohorts were included: MTX-starters = Rheumatoid Arthritis Medication Study; and biologic-starters = Biologics in Rheumatoid Arthritis Genetics and Genomics Study Syndicate. Presenteeism and absenteeism were assessed using the RA-specific Work Productivity Survey at baseline, and 6 and 12 months. Potential predictors including baseline age, gender, clinical measures (e.g. disability, pain, fatigue), psychological distress, occupation and EULAR response from baseline to 6 months were investigated.

**Results:**

A total of 51/463 MTX-starters and 30/260 biologic-starters left work over 12 months. Higher baseline psychological distress in MTX-starters [odds ratio (OR) 1.1 (95% CI: 1.0, 1.1)] and higher disability in biologic-starters [OR 3.5 (95% CI: 1.4, 8.6)] predicted work loss. Some 16.1% of patients reported sick-leave, which was predicted by disability [OR (95% CI): MTX-starters: 1.5 (0.9, 2.3); biologic-starters: 2.4 (1.1, 5.2)]. Median presenteeism scores were very low (minimal interference) in both cohorts. Higher fatigue for MTX starters [incidence rate ratio 1.2 (95% CI: 1.0, 1.4)] and higher disability in biologic-starters (incidence rate ratio 1.4 (95% CI: 1.1, 1.7)] predicted presenteeism. Good EULAR response was associated with lower absenteeism and presenteeism in both cohorts.

**Conclusion:**

Patients with RA still face significant limitations regarding their ability to work. Disability and EULAR response were the main predictors of work outcomes, emphasizing the need to control the disease and the importance of function in enabling work participation.

**Rheumatology key messages**

- Patients with RA still face significant work limitation in the modern treatment era.
- Disability, fatigue and psychological distress were identified as key predictors of work outcomes.
- Disease management may require a holistic approach, alongside good disease control, to improve work outcomes.

## Introduction

Work is important for quality of life, economic independence and a sense of purpose [1]. Patients with RA consistently report that they want to remain in work, but research has shown that 36–84% of individuals with RA take sickness absence because of their condition (absenteeism), and up to 50% of patients have to stop work altogether over a period of 4.5–22 years [2]. In the UK, 10% of early RA patients left work over a median of 3 years of follow-up [3], and 49% of patients starting a biologic were work disabled [4]. This loss of productivity is accompanied by significant socio-economic consequences [5–7]. Attempts made by employers and policy makers to reduce absenteeism and maintain work retention may lead to an increased likelihood of reduced job performance at work (known as presenteeism) [8]. Presenteeism is common in all workforces (everybody performs below their peak on occasion because of stress, aches and pains, or mild infections) and is not universally undesirable. For example, a phased return to work after prolonged sickness absence is a desirable form of presenteeism aiming towards total rehabilitation. However, presenteeism can be harmful to the individual and can increase demands on co-workers, and thus the factors associated with presenteeism are receiving increased research attention [9].

Historically, the majority of research exploring factors associated with work participation in RA has concentrated on job loss (e.g. [10–12]), and to a lesser extent absenteeism and presenteeism. Cross-sectional studies have reported that higher disease activity, higher disability, poorer mental health and lower quality of life are associated with presenteeism and absenteeism [13–15]. Longitudinal predictors are less well studied but, amongst patients with early RA, presenteeism was higher in those with physically demanding jobs and those reporting less support from colleagues [16, 17]. In patients with established RA, greater disability and poorer mental health predicted presenteeism over 1 year [18].

A weakness of existing research on work participation in RA cohorts is the inclusion of a wide range of symptom durations. Therefore, we investigated baseline factors associated with three important work measures—job loss, absenteeism and presenteeism—over 12 months of follow-up in two cohorts of patients at fixed points in the disease progression of RA: at the initiation of MTX and at the initiation of biologic therapy.

## Methods

### Patients and setting

Participants were recruited to one of two UK multicentre 1 year prospective observational studies: the Rheumatoid Arthritis Medication Study (RAMS) (MTX-starters), or the Biologics in Rheumatoid Arthritis Genetics and Genomics Study Syndicate (BRAGGSS) (biologic-starters). Recruiting centres were spread across the UK, including both teaching and non-teaching centres as well as serving both urban and rural populations. The detailed methods of both studies have been published elsewhere [19, 20]. In brief, the studies recruited patients with RA starting either MTX or biologics, collecting data at baseline (treatment initiation), and 6 and 12 months. The inclusion criteria for the current analysis were: aged 18–65 years, in paid full- or part-time employment at the start of treatment, and employment data for at least one follow-up. MTX-starters were recruited from 2008; those with >2 years symptom duration were excluded from this analysis. Biologic-starters were limited to those starting their first biologic. Biologic-starters were recruited from 2008. However, work outcomes were only collected from 2012 amongst biologic-starters; therefore, only biologic-starters recruited after 2012 were included.

All patients gave written informed consent. Ethical approval for the MTX-starters cohort was obtained from the National Research Ethics Service Central Manchester Research Ethics Committee (ref.: 08/H1008/25) and ethical approval for the biologic-starters cohort came from the North West Ethics Committee (ref.: 04/Q1403/37).

### Clinical data collection

Age, gender, symptom duration and smoking status were collected at baseline. The DAS28 and its components [28 swollen and tender joint counts; CRP (mg/l; assayed at central biocentre); patient global visual analogue scale (100 mm VAS)] were assessed at each assessment [21]. EULAR response (good, moderate, no response) from baseline to 6 months was calculated [22]. Comorbidities were self-reported by patients from a pre-defined list, and were categorized for analysis as: no comorbidities; 1 comorbidity; ≥2 comorbidities.

Patients completed a questionnaire at each visit measuring pain and fatigue (0–100 mm VAS where high scores indicate worse state), functional ability [HAQ (British version)] [23], quality of life (assessed using the EQ5D with scores calculated using the Time Trade-off valuation technique) [24], and anxiety and depression [Hospital Anxiety and Depression Scale (HADS); higher scores are worse] [25].

### Work measures

Patients recorded their current work status (full-time, part-time, work disabled, retired, retired early due to arthritis, unemployed, working full time in the home) at baseline and subsequent assessments. Patients were coded as in work if they reported being in paid work (part-time or full-time) or on temporary sick-leave, and as having left the labour force if they reported any other work status at follow-up. Self-reported occupation was coded using the UK Standard Occupational Classification [26, 27]. These codes were then used to classify jobs into three classes using the National Statistics Socio-Economic Classification (NS-SEC) [28]. Absenteeism (sick-leave) was measured as days missed from work in the last month due to RA. Due to the extremely high number of zeros (MTX-starters = 82.4%; biologic-starters = 85.7%) and sparse data for each of the other possible values (1–31 days), absenteeism data were dichotomized into reported any sick-leave, yes or no. Everybody completed an adapted version of the RA-specific Work Productivity Survey [9, 29–31], which measures presenteeism with the question ‘In the last month, how much has arthritis interfered with your work productivity (paid work) on a scale of 0–10, where 0 = no interference and 10 = complete interference’.

### Statistical analysis

Descriptive statistics were used to summarize the baseline and work outcome data for each cohort. Candidate predictors were selected *a priori* to be assessed in univariable analyses as follows: age, gender, smoking status, swollen joint count (SJC), HAQ, pain-VAS, fatigue-VAS, HADS Depression, HADS Anxiety, EQ5D, NS-SEC and comorbidities. The baseline EQ5D, pain-VAS and fatigue-VAS were standardized, meaning that these coefficients represent a change in the outcome for a 1 s.d. change of the baseline factor. Due to the relatively low number of patients leaving work and taking sick leave, some modelling decisions were made when constructing multivariable models based on univariable analysis and theory, namely: EQ5D was excluded due to high collinearity with other variables, HADS Depression and Anxiety were combined to produce a psychological distress score [32] and SJC was removed after univariable analysis. Patients who reported having left work at an assessment did not contribute to the absenteeism and presenteeism analyses at that or subsequent assessments, but did contribute to these outcomes at earlier time-points. Predictors of leaving work and absenteeism were assessed using population average logistic regression models—a type of longitudinal model that included data from both time-points and adjusts for within-patient correlation. Odds ratios (ORs) are reported. A zero-inflated negative binomial regression model was used to model presenteeism due to excess zeros, with standard errors adjusted for within-patient clustering over the repeated measures. Zero-inflated negative binomial regression models assume that a zero score and a count score are produced by two separate processes. A logit model is used to predict whether a participant belongs in the zero presenteeism group (i.e. predictors of no presenteeism; ORs reported), and a negative binomial model predicts the count data [i.e. amount of presenteeism if presenteeism occurred; incidence rate ratios (IRRs) reported]. EULAR response (good, moderate, none) at 6 months was used to predict work participation over follow-up using population average logistic and negative binomial regression, controlling for age and gender. Those in remission at baseline were excluded from the EULAR response analysis (i.e. DAS28 <2.6). Multiple imputation was used to account for missing baseline data [10 imputed datasets created; variables ranged from complete to 15.4% missing (NS-SEC in biologic-starters cohort)]. Statistical analyses were performed using Stata 14 (StataCorp, College Station, TX, USA).

## Results

In total, 463 (43.5%) of 1065 18- to 65-year-old MTX-starters and 260 (20.9%) of 1247 18- to 65-year-old biologic-starters met the inclusion criteria and were available for these analyses. The median age in both cohorts was 52 years (interquartile range: MTX-starters = 45, 57; biologic-starters = 45.5, 57). There was a higher proportion of women in the biologic-starters compared with MTX-starters [MTX-starters = 315 (68.0%); biologic-starters = 201 (77.3%)]. As expected, symptom duration was longer in biologic-starters [median (interquartile range) months: MTX-starters = 7 (4, 12); biologic-starters = 48 (24, 102)]. Biologic-starters also had higher median scores for all disease activity measures and patient-reported outcomes compared with MTX-starters (Table 1). Of those with sick-leave data at baseline, 107/350 (30.6%) MTX-starters and 53/208 (25.5%) biologic-starters reported taking sick-leave in the month prior to baseline.

**Table 1:** Baseline characteristics of the two cohorts

	Total cohort				Patients included in work analyses			
	MTX-starters		Biologic-starters		MTX-starters		Biologic-starters	
Variable	*N* (%)	Median (IQR)	*N* (%)	Median (IQR)	*N* (%)	Median (IQR)	*N* (%)	Median (IQR)
Age at baseline, years	1065	52 (44, 59)	1247	54 (47, 59)	463	52 (45, 57)	260	52 (45.5, 57)
Female (% of total cohort)	742 (69.7)		970 (77.9)		315 (68.0)		201 (77.3)	
Symptom duration, months	1065	7 (4, 12)	1233	60 (24, 120)	463	7 (4, 12)	256	48 (24, 102)
Smoking status								
Never	407 (38.5)		452 (36.6)		190 (41.1)		102 (39.2)	
Former	374 (35.4)		504 (40.8)		175 (37.9)		113 (43.5)	
Current	276 (26.1)		280 (22.7)		97 (21.0)		45 (17.3)	
SJC (28 joints)	1020	4 (2, 9)	1190	8 (5, 11)	448	4 (1, 8)	250	8 (5, 12)
Tender joint count (28 joints)	1019	6 (2, 13)	1190	15 (10, 21)	449	5 (2, 11)	249	14 (9, 20)
CRP (mg/l)	1056	5 (2, 14)	1027	9 (4, 22)	461	5 (2, 12)	227	8 (4, 17)
DAS28-CRP	1000	4.3 (3.2, 5.2)	995	5.8 (5.2, 6.3)	442	4.0 (3.1, 4.9)	219	5.6 (5.2, 6.2)
HAQ	957	1.00 (0.38, 1.50)	944	1.63 (1.13, 2.13)	461	0.88 (0.38, 1.38)	258	1.38 (0.88, 1.75)
RF status								
* Total measured*	818		1058		384		224	
* Positive*	553 (67.6)		691 (65.3)		277 (72.1)		154 (68.8)	
Pain-VAS	946	51 (28, 72)	929	70 (54, 81)	459	48 (25, 68)	256	67 (49, 78)
Fatigue-VAS	945	57 (29, 76)	927	76 (60, 88)	458	53 (25, 73)	256	72 (52, 85)
Patient global VAS	1056	43 (22, 64)	1177	80 (65, 90)	459	35 (20, 60)	244	78 (65, 86)
HADS Depression	953	6 (2, 9)	920	7.5 (5, 11)	461	5 (2, 8)	256	6 (3, 9)
HADS Anxiety	952	6.5 (4, 10)	914	8 (5, 12)	460	6 (3, 9)	256	8 (5, 11)
EQ-5D	935	0.66 (0.52, 0.76)	911	0.52 (-0.02, 0.66)	453	0.69 (0.52, 0.76)	254	0.59 (0.19, 0.69)
Comorbidity								
No comorbidity	501 (47.0)		418 (39.9)		236 (51.0)		128 (53.3)	
One comorbid condition	355 (33.3)		369 (35.2)		155 (33.5)		77 (32.1)	
≥2 comorbid conditions	209 (19.6)		261 (24.9)		72 (15.6)		35 (14.6)	

DAS28-CRP: DAS28 using CRP; HADS: Hospital Anxiety and Depression Scales; IQR: interquartile range; *N*: number; VAS: visual analogue scale.

### Baseline occupation

The occupations of MTX-starters were evenly spread across the three NS-SEC classes, with around a third in each class. There was a slightly higher proportion of biologic-starters in NS-SEC class one, representing higher managerial jobs (Table 2) (supplementary Table S1, available at *Rheumatology* online for distribution across the nine UK Standard Occupational Classification chapters). At baseline, 87 (19.2%) MTX-starters and 121 (47.8%) biologic-starters reported having made adaptions to their work environment since symptom onset.

**Table 2:** The NS-SEC classes of the two cohorts

NS-SEC classes	MTX-starters, *N* (%)	Biologic-starters, *N* (%)
Class 1: Higher managerial, administrative and professional occupations	160 (34.6)	81 (31.2)
Class 2: Intermediate occupations	144 (31.1)	70 (26.9)
Class 3: Routine and manual occupations	143 (30.9)	69 (26.5)
Uncoded^a^	16 (3.5)	40 (15.4)

a Patients who did not provide enough information on their occupation to be coded using the UK Standard Occupational Classification. *N*: number; NS-SEC: The National Statistics Socio-Economic Classification.

### Work status over 1 year

**Fig. 1:**
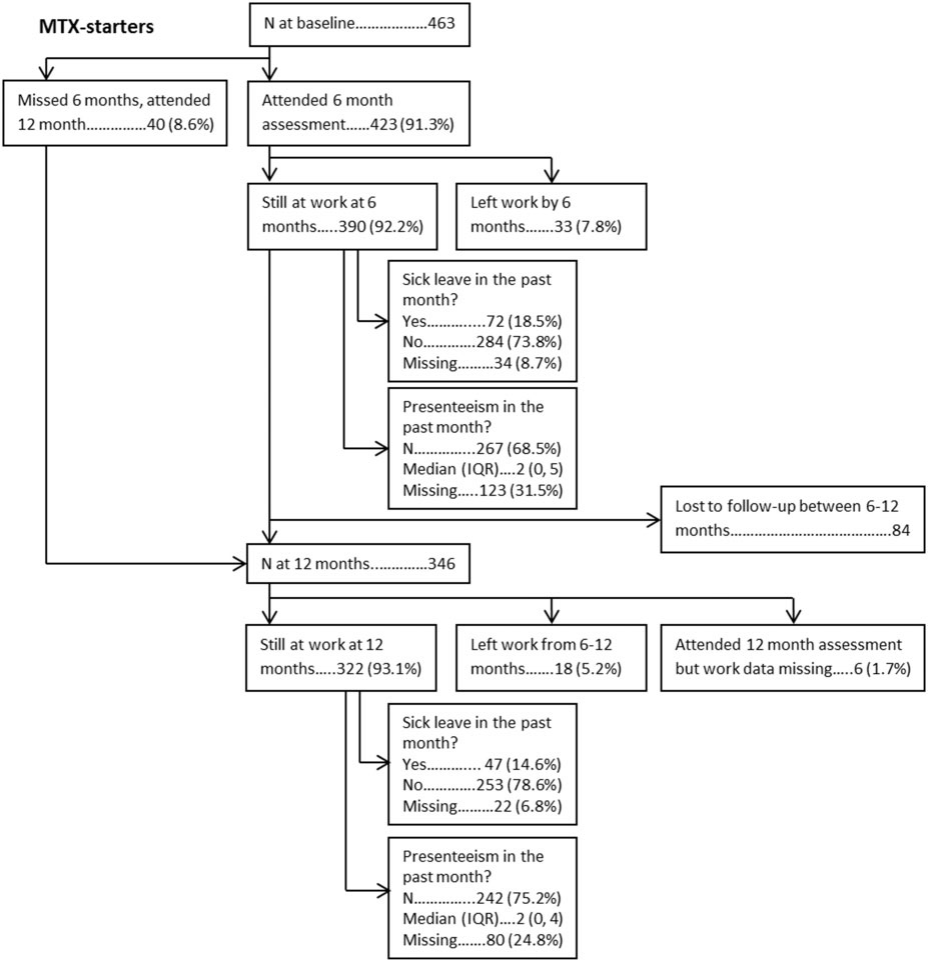
Flowchart and work outcomes (MTX-starters)

**Fig. 2:**
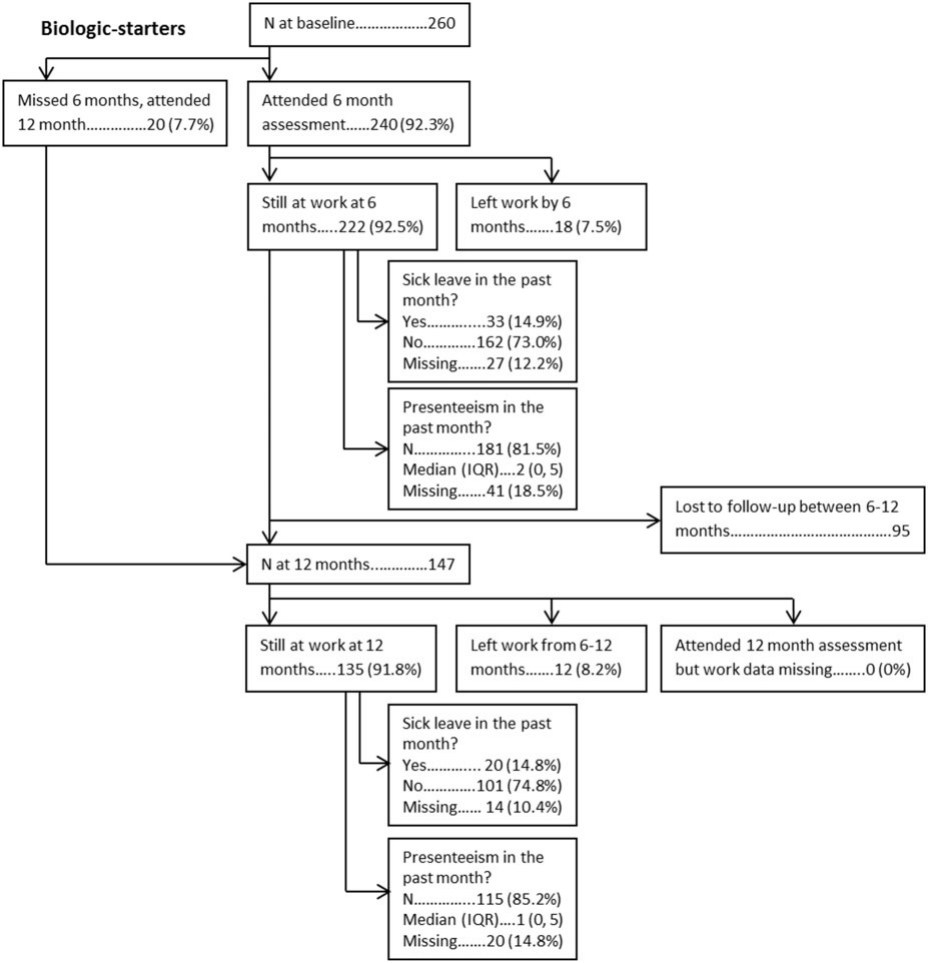
Flowchart and work outcomes (biologic-starters)

From baseline to 6 months, 33/423 (7.8%) MTX-starters and 18/240 (7.5%) biologic-starters left work. From 6 to 12 months, 18/346 (5.2%) MTX-starters and 12/147 (8.2%) biologic-starters left work. Twenty patients became work disabled (MTX-starters = 15; biologic-starters = 5), 17 retired (MTX-starters = 9; biologic-starters = 8), 13 retired early due to arthritis (MTX-starters = 6; biologic-starters = 7) and 31 left for other reasons (MTX-starters = 21; biologic-starters = 10). Of those still working, 72/356 (20.2%) and 47/300 (15.6%) MTX-starters took sick-leave the month preceding their 6 and 12 month assessment respectively. For biologic-starters, the proportions were similar [6 months = 33/195 (16.9%); 12 months = 20/121 (16.5%)]. For both cohorts, the proportions taking sick-leave at 6 and 12 months were lower than the proportions at baseline. Low rates of presenteeism were reported by patients at both assessments in both cohorts (6 months, median (interquartile range): MTX-starters = 2 (0, 5), biologic-starters = 2 (0, 5); 12 months: MTX-starters = 2 (0, 4), biologic-starters = 1 (0, 5)] (Figs 1 and 2).

### Baseline predictors of work outcomes

There were several significant univariable baseline predictors of leaving work in both cohorts (MTX-starters: current smoking, HAQ, HADS Depression, HADS Anxiety, EQ5D, NS-SEC; biologic-starters: age) (Table 3). In multivariable analysis, the odds of leaving work increased by 55% per unit increase in disability (HAQ: OR 1.55, 95% CI: 0.85, 2.84) for MTX starters. General psychological distress predicted leaving work amongst MTX-starters (OR 1.05, 95% CI: 1.00, 1.10), as did current smoking (*vs* never smoking: OR 3.47, 95% CI: 1.65, 7.31), and people in routine and manual occupations were more likely to leave work compared with those in higher managerial occupations (OR 2.14, 95% CI: 1.01, 4.54). For biologic-starters, more functional disability predicted leaving work (OR per unit increase in HAQ: 3.46, 95% CI: 1.39, 8.62) (Table 4).

**Table 3:** Predictors of work outcomes from univariable models

	Left work, OR (95% CI)		Sick-leave, OR (95% CI)		No presenteeism, OR (95% CI)		Presenteeism Score, IRR (95% CI)	
Baseline predictor	MTX-starters (*N* = 463)	Biologic-starters (*N* = 260)	MTX-starters (*N* = 404)	Biologic-starters (*N* = 217)	MTX-starters (*N* = 325)	Biologic-starters (*N* = 207)	MTX-starters (*N* = 325)	Biologic-starters (*N* = 207)
Age	1.01 (0.97, 1.04)	1.05 (1.00, 1.11)	0.98 (0.96, 1.00)	1.00 (0.97, 1.04)	1.01 (0.98, 1.05)	1.01 (0.99, 1.04)	1.00 (0.99, 1.01)	1.00 (0.99, 1.01)
Women *vs* men	1.18 (0.63, 2.19)	0.76 (0.32, 1.77)	1.57 (0.95, 2.61)	0.94 (0.43, 2.05)	1.00 (0.56, 1.79)	0.97 (0.52, 1.80)	1.43 (1.16, 1.77)	1.10 (0.85, 1.41)
Smoking vs never								
Former	1.10 (0.51, 2.35)	0.94 (0.41, 2.16)	0.84 (0.51, 1.37)	1.32 (0.64, 2.69)	0.76 (0.42, 1.35)	1.45 (0.80, 2.64)	0.93 (0.74, 1.21)	1.15 (0.92, 1.42)
Current	3.78 (1.88, 7.60)	1.25 (0.45, 3.49)	1.09 (0.59, 1.99)	1.58 (0.64, 3.91)	0.48 (0.20, 1.15)	1.19 (0.53, 2.68)	0.94 (0.74, 1.21)	1.21 (0.91, 1.63)
SJC28	1.02 (0.97, 1.07)	1.02 (0.94, 1.10)	1.01 (0.97, 1.05)	1.00 (0.94, 1.07)	1.00 (0.95, 1.06)	1.09 (1.03, 1.16)	1.01 (0.99, 1.03)	1.01 (0.99, 1.03)
HAQ	1.63 (1.08, 2.46)	1.83 (0.96, 3.49)	2.08 (1.50, 2.89)	2.86 (1.63, 5.05)	0.43 (0.28, 0.68)	0.47 (0.29, 0.76)	1.37 (1.22, 1.54)	1.55 (1.30, 1.86)
Pain-VAS								
Natural scale	1.01 (0.995, 1.02)	0.99 (0.98, 1.01)	1.02 (1.01, 1.03)	1.01 (0.998, 1.03)	0.98 (0.97, 1.00)	0.99 (0.98, 1.01)	1.01 (1.00, 1.01)	1.01 (1.00, 1.01)
Standardized scale	1.18 (0.88, 1.57)	0.83 (0.58, 1.18)	1.53 (1.22, 1.93)	1.38 (0.97, 1.95)	0.65 (0.47, 0.90)	0.88 (0.66, 1.17)	1.23 (1.11, 1.36)	1.16 (1.03, 1.30)
Fatigue-VAS								
Natural scale	1.01 (0.997, 1.02)	1.00 (0.98, 1.01)	1.02 (1.01, 1.03)	1.02 (1.00, 1.03)	0.97 (0.96, 0.98)	0.99 (0.98, 1.00)	1.01 (1.01, 1.01)	1.01 (1.00, 1.02)
Standardized scale	1.22 (0.91, 1.64)	0.90 (0.63, 1.30)	1.80 (1.41, 2.31)	1.50 (1.03, 2.19)	0.46 (0.34, 0.63)	0.83 (0.63, 1.10)	1.31 (1.19, 1.45)	1.26 (1.10, 1.45)
HADS Depression	1.11 (1.03, 1.19)	1.05 (0.95, 1.15)	1.13 (1.07, 1.19)	1.14 (1.05, 1.23)	0.83 (0.75, 0.92)	0.89 (0.82, 0.96)	1.06 (1.03, 1.08)	1.04 (1.02, 1.06)
HADS Anxiety	1.12 (1.05, 1.19)	1.04 (0.95, 1.14)	1.12 (1.06, 1.18)	1.09 (1.01, 1.18)	0.85 (0.78, 0.93)	0.88 (0.82, 0.95)	1.04 (1.02, 1.06)	1.03 (1.01, 1.05)
EQ5D (standardized)	0.74 (0.58, 0.96)	0.82 (0.57, 1.17)	0.61 (0.50, 0.75)	0.61 (0.45, 0.83)	1.94 (1.24, 3.02)	1.46 (1.08, 1.98)	0.80 (0.74, 0.87)	0.88 (0.80, 0.96)
NS-SEC								
Class 2 *vs* class 1	1.22 (0.55, 2.71)	0.79 (0.30, 2.06)	0.68 (0.38, 1.22)	1.89 (0.84, 4.29)	1.32 (0.70, 2.51)	0.94 (0.44, 2.02)	0.79 (0.62, 0.99)	1.31 (1.03, 1.67)
Class 3 *vs* class 1	2.22 (1.05, 4.70)	0.62 (0.24, 1.64)	1.32 (0.78, 2.23)	1.89 (0.84, 4.28)	0.93 (0.48, 1.80)	1.45 (0.72, 2.90)	0.99 (0.80, 1.21)	1.31 (1.02, 1.69)
Comorbidity								
1 comorbidity *vs* 0	0.81 (0.41, 1.60)	1.45 (0.61, 3.42)	1.19 (0.73, 1.94)	1.25 (0.60, 2.59)	0.79 (0.44, 1.42)	0.72 (0.38, 1.35)	1.11 (0.91, 1.36)	1.03 (0.81, 1.31)
≥2 comorbidities *vs* 0	1.62 (0.79, 3.35)	1.81 (0.64, 5.16)	1.33 (0.71, 2.49)	1.51 (0.58, 3.94)	0.66 (0.27, 1.62)	0.37 (0.16, 0.86)	1.17 (0.91, 1.50)	1.18 (0.88, 1.56)

The ORs and IRRs are interpreted as the change in odds or the relative change in the outcome per unit increase in the predictor. For the natural scale of the Pain- and Fatigue-VAS, a 1 unit change is very small. Hence, the results are also given on a standardized scale. Here, a 1 unit increase in the scale corresponds to 1 s.d. change in the visual analogue scale score (approx. 20 unit change in this case). Interpretation of ZINB regression output used to model presenteeism scores: ZINB models data with a high number of zeros (i.e. presenteeism) by splitting the data into two portions, the excess zero scores and the count scores. Then the model predicts whether or not a patient had an excess zero for presenteeism or not using a logistic regression model, and gives OR for no presenteeism (with a score <1 indicating that, as the variable increases, patients are less likely to have no presenteeism). After this, the model predicts the count scores, using a negative binomial regression model and gives IRRs (with a score >1 indicating that as the predictor increases, presenteeism score increases; for example, an IRR of 1.55 for HAQ scores of biologic-starters indicates that, for each unit increase in HAQ score, the average presenteeism score increases by 55%). HADS: Hospital Anxiety and Depression Scale; IRR: incidence rate ratio; NS-SEC: The National Statistics Socio-Economic Classification (see Table 2 for definition of classes); OR: odds ratio; SJC: swollen joint count; VAS: visual analogue scale; Natural scale: the unadjusted scores from the visual analogue scales ranging from 0–100; ZINB: zero-inflated negative binomial.

**Table 4:** Independent predictors of work outcomes from multivariable models

	Left work, OR (95% CI)		Sick-leave, OR (95% CI )		No presenteeism, OR (95% CI)		Presenteeism Score, IRR (95% CI)	
Baseline predictor	MTX-starters (*N* = 463)	Biologic-starters (*N* = 260)	MTX-starters (*N* = 404)	Biologic-starters (*N* = 217)	MTX-starters (*N* = 325)	Biologic-starters (*N* = 207)	MTX-starters (*N* = 325)	Biologic-starters (*N* = 207)
AgeAQ10	1.00 (0.96, 1.03)	1.04 (0.99, 1.11)	0.98 (0.96, 1.01)	1.00 (0.96, 1.04)	1.02 (0.98, 1.06)	1.02 (0.99, 1.05)	1.00 (0.99, 1.01)	1.00 (0.99, 1.02)
Women *vs* men	1.31 (0.66, 2.63)	0.79 (0.32, 2.00)	1.21 (0.71, 2.08)	0.68 (0.30, 1.56)	1.40 (0.63, 3.08)	1.45 (0.76, 2.78)	1.27 (1.01, 1.60)	1.03 (0.79, 1.34)
Smoking status *vs* never								
Former	1.01 (0.45, 2.24)	0.66 (0.27, 1.63)	0.83 (0.49, 1.40)	1.08 (0.51, 2.28)	0.66 (0.34, 1.27)	1.74 (0.89, 3.42)	0.94 (0.78, 1.13)	1.10 (0.89, 1.36)
Current	3.47 (1.65, 7.31)	0.84 (0.28, 2.54)	1.01 (0.53, 1.93)	1.02 (0.38, 2.70)	0.44 (0.16, 1.21)	1.58 (0.63, 3.95)	0.96 (0.77, 1.20)	0.95 (0.70, 1.28)
HAQ	1.55 (0.85, 2.84)	3.46 (1.39, 8.62)	1.46 (0.90, 2.34)	2.41 (1.12, 5.22)	0.63 (0.34, 1.18)	0.42 (0.21, 0.84)	1.08 (0.92, 1.28)	1.40 (1.13, 1.74)
Pain-VAS								
Natural scale	1.00 (0.98, 1.01)	0.98 (0.96, 1.00)	1.00 (0.99, 1.01)	1.00 (0.98, 1.01)	1.01 (0.99, 1.03)	1.01 (0.99, 1.03)	1.00 (0.997, 1.01)	1.00 (0.99, 1.00)
Standardized scale	0.90 (0.58, 1.39)	0.65 (0.41, 1.04)	0.96 (0.68, 1.35)	0.90 (0.59, 1.38)	1.33 (0.83, 2.11)	1.28 (0.85, 1.93)	1.05 (0.92, 1.20)	0.99 (0.88, 1.12)
Fatigue-VAS								
Natural scale	1.00 (0.98, 1.01)	0.99 (0.97, 1.01)	1.01 (0.999, 1.02)	1.00 (0.98, 1.02)	0.98 (0.96, 1.00)	1.01 (0.99, 1.02)	1.01 (1.00, 1.01)	1.01 (0.999, 1.01)
Standardized scale	0.88 (0.56, 1.35)	0.71 (0.42, 1.19)	1.35 (0.97, 1.89)	1.05 (0.66, 1.69)	0.54 (0.34, 0.86)	1.20 (0.81, 1.78)	1.17 (1.01, 1.35)	1.15 (0.98, 1.34)
HADS General distress	1.05 (1.00, 1.10)	1.04 (0.97, 1.11)	1.04 (1.00, 1.08)	1.04 (0.98, 1.09)	0.95 (0.88, 1.02)	0.94 (0.89, 0.99)	1.01 (0.99, 1.02)	1.00 (0.99, 1.02)
NS-SEC								
Class 2 *vs* class 1	1.28 (0.56, 2.92)	0.57 (0.20, 1.65)	0.67 (0.37, 1.21)	1.58 (0.66, 3.80)	1.11 (0.53, 2.36)	1.12 (0.48, 2.63)	0.85 (0.69, 1.05)	1.22 (0.97, 1.55)
Class 3 *vs* class 1	2.14 (1.01, 4.54)	0.46 (0.15, 1.37)	1.39 (0.80, 2.40)	1.54 (0.65, 3.65)	0.92 (0.45, 1.87)	2.08 (0.90, 4.80)	1.07 (0.89, 1.29)	1.20 (0.94, 1.53)
Comorbidity								
1 comorbidity *vs* 0	0.81 (0.40, 1.66)	0.89 (0.34, 2.29)	1.16 (0.70, 1.92)	0.97 (0.43, 2.16)	0.92 (0.49, 1.73)	0.82 (0.39, 1.71)	1.03 (0.85, 1.23)	1.05 (0.83, 1.33)
≥2 comorbidities *vs* 0	1.58 (0.72, 3.48)	1.12 (0.33, 3.76)	1.06 (0.55, 2.07)	0.92 (0.32, 2.66)	0.99 (0.37, 2.68)	0.36 (0.13, 0.98)	1.01 (0.79, 1.28)	1.01 (0.77, 1.33)

HADS: Hospital Anxiety and Depression Scale; IRR: incidence rate ratio; NS-SEC: The National Statistics Socio-Economic Classification (see Table 2 for definition of classes); OR: odds ratio; VAS: visual analogue scale; Natural scale: the unadjusted scores from the visual analogue scales ranging from 0–100.

Amongst those remaining in the work-force, there were a number of univariable baseline predictors of absenteeism (MTX-starters: age, HAQ, pain-VAS, fatigue-VAS, HADS Depression, HADS Anxiety, EQ5D; biologic-starters: HAQ, fatigue-VAS, HADS Depression, HADS Anxiety, EQ5D) (Table 3). In multivariable analysis, more functional disability (OR per unit increase in HAQ: 1.46, 95% CI: 0.90, 2.34), more fatigue (OR per s.d. increase in fatigue-VAS: 1.35, 95% CI: 0.97, 1.89) and greater psychological distress (OR per unit increase in HADS psychological distress: 1.04, 95% CI: 1.00, 1.08) predicted absenteeism for MTX-starters (Table 4). Amongst biologic-starters, more disability predicted absenteeism (OR per unit increase in HAQ: 2.41, 95% CI: 1.12, 5.22) (Table 4).

There were several univariable baseline predictors of reporting no presenteeism from the zero-inflated negative binomial analyses (MTX-starters: HAQ, pain-VAS, fatigue-VAS, HADS Depression, HADS Anxiety, EQ5D; biologic-starters: SJC28, HAQ, HADS Depression, HADS Anxiety, EQ5D) (Table 3). Baseline independent predictors of no presenteeism in MTX-starters were less fatigue (OR per s.d. increase in fatigue-VAS: 0.54, 95% CI: 0.34, 0.86) and less disability (OR per unit increase in HAQ: 0.63, 95% CI: 0.34, 1.18). For biologic-starters, less disability (OR per unit increase in HAQ: 0.42, 95% CI: 0.21, 0.84), less psychological distress (OR per unit increase in HADS psychological distress: 0.94, 95% CI: 0.89, 0.99) and being in routine and manual occupations *vs* higher managerial, administrative and professional occupations (OR 2.08, 95% CI: 0.90, 4.80) predicted no presenteeism (Table 4). Of the patients with presenteeism, independent baseline predictors of higher presenteeism score in MTX-starters included: female gender (IRR women *vs* men: 1.27, 95% CI: 1.01, 1.60) and more fatigue (IRR per s.d. increase in fatigue-VAS: 1.17, 95% CI: 1.01, 1.35). For biologic-starters, higher baseline disability (IRR per unit increase in HAQ: 1.40, 95% CI: 1.13, 1.74) and higher baseline fatigue (IRR per s.d. increase in fatigue-VAS: 1.15, 95% CI: 0.98, 1.34) predicted more presenteeism over follow-up.

### EULAR response over the first 6 months and work outcomes

In total, 121 (31.4%) MTX-starters and 89 (57.8%) biologic-starters had a good response and 99 (25.7%) MTX-starters and 49 (31.8%) biologic-starters had moderate response over the first 6 months. EULAR response was not significantly associated with leaving the labour force over follow-up in MTX-starters (moderate *vs* no response: OR 1.30, 95% CI: 0.60, 2.85; good *vs* no response: OR 0.90, 95% CI: 0.41, 1.93) or biologic-starters (moderate *vs* no response: OR 0.88, 95% CI: 0.23, 3.36; good *vs* no response: OR 0.57, 95% CI: 0.16, 2.09). However amongst MTX-starters, both moderate and good response were associated with lower odds of absenteeism (MTX-starters: moderate *vs* no response: OR 0.36, 95% CI: 0.19, 0.67; good *vs* no response: OR 0.23, 95% CI: 0.12, 0.42) and less presenteeism (MTX-starters: moderate *vs* no response: IRR 0.71, 95% CI: 0.50, 1.00; good *vs* no response: IRR 0.38, 95% CI: 0.27, 0.52) compared with no response. For biologic-starters, good response was associated with lower odds of absenteeism (biologic-starters: moderate *vs* no response: OR 0.44, 95% CI: 0.14, 1.34; good *vs* no response: OR 0.36, 95% CI: 0.13, 1.04) and less presenteeism compared with no response (biologic-starters: moderate *vs* no response: IRR 0.72, 95% CI: 0.41, 1.26; good *vs* no response: IRR 0.44, 95% CI: 0.27, 0.71); the effect sizes of the moderate *vs* no response comparisons also indicated an association in favour of moderate response, but the CI overlapped one in both analyses.

## Discussion

These analyses of over 700 people with RA in paid employment when starting either MTX or a biologic have allowed us to explore the prevalence of and risk factors for job loss, sickness absence and presenteeism over 12 months of follow-up. Overall, 11% of the MTX-starters and 11.5% of biologic-starters left work during the study period. On average, 16.6% of MTX-starters and 14.9% of biologic-starters took sickness absence in the month prior to each follow-up, a reduction from baseline. The reported rates of presenteeism were low in this study. Higher ratings of disability at baseline predicted poorer work outcomes, highlighting the importance of function in enabling work participation. Fatigue, psychological distress and job type also significantly predicted different work outcomes. Furthermore, good treatment response was associated with lower odds of absenteeism and lower presenteeism scores. Therefore, a holistic approach combining both good control of disease activity and interventions aiming to address the other salient predictors including reducing physical demands and increasing support at work, and improving disability and fatigue, may be required to further improve the work participation of patients with RA.

Over 10% of patients with RA starting MTX left work over the first year, rates similar to those in another UK cohort recruited in the 2000s [3]. Whilst these rates are lower than those reported in a UK study of early RA patients recruited in the 1990s [33], this represents significant loss to the work force and a major negative impact on these patients’ lives. Disability, current smoking and psychological distress predicted leaving work for MTX-starters, as did manual occupations [3, 4, 10, 34–39]. Biologic treatment has been shown to have positive effects on work participation in the past [40], and a similar proportion of biologic-starters left work compared with MTX-starters. In the current study, biologic-starters differ from MTX-starters as they have remained in work for longer since the onset of RA. Patients at high risk of leaving work may have done so prior to starting a biologic. This attrition effect may explain why psychological distress and smoking no longer predict leaving work in biologic-starters. However, disability still predicted leaving work in biologic-starters, indicating the importance of monitoring and responding to other aspects of RA symptomology beyond disease activity. Interestingly, lower baseline pain also predicted biologic-starters leaving work and we found an interaction in this group between pain and age, whereby lower pain predicted leaving work in older patients only, although a low number of events meant that this interaction was not statistically significant and may be a chance finding (data not shown).

Of those remaining in work after starting MTX, around 20% of patients reported taking sick-leave in the months preceding each assessment. These levels of absenteeism are similar to those found in other observational studies, although comparisons are difficult since methods for collecting absenteeism data are not standardized [41]. Higher fatigue, psychological distress and disability predicted taking sick-leave for MTX-starters. Fatigue has been reported elsewhere as a key factor affecting the ability to remain in work with RA [42, 43]. Similar proportions of biologic-starters reported taking sick leave. In these patients, disability was the only significant predictor of taking sick-leave [38]. The HAQ assesses difficulties performing activities of daily living and clearly points to the importance of function in work participation.

Presenteeism scores were low in both cohorts, as found elsewhere [41]. Whilst this may suggest that productivity is not affected by arthritis when patients are able to attend work, there are concerns regarding the utility of a 10-point scale in detecting presenteeism accurately. In both cohorts, fatigue, psychological distress and disability predicted presenteeism [16, 38, 41, 44]; for biologic-starters, presenteeism was less likely amongst those with fewer comorbidities.

Interventions to improve work outcomes have been developed that examine work schedules and environments, and barriers to work [45–47], although with uncertain efficacy [48]. Targeted referral to interventions aiming to address important areas influencing patients’ personal ability to work (e.g. disability, fatigue, psychological distress) for patients at high risk of job loss may be the next step towards improving work participation amongst patients with RA.

This analysis has a number of strengths. This study combines two large cohorts with similar methods and assessments but recruiting patients at different stages of disease progression, allowing comparison of prognostic factors across RA disease progression. Limitations of the study include the fact that productivity loss is self-reported, meaning we are unable to assess objective reductions in productivity. Furthermore, the measure used to detect presenteeism may not be a sensitive tool for measuring this multi-dimensional concept, although the tool has been shown to have good validity and reliability in the past [30, 31]. There were also missing data for presenteeism scores in both cohorts, with some differences in baseline factors. However, analysis using imputed presenteeism scores did not differ substantially from the unimputed outcome analysis (see supplementary Tables S2 and S3, available at *Rheumatology* online).

In conclusion, there are a number of important baseline factors that predict different work-related outcomes. These factors may be useful in identifying patients who are at increased risk of having poor work-related outcomes. This indicates that a holistic approach towards disease management is necessary, alongside good control of disease activity, to improve the work-related outcomes of patients with RA.

## Supplementary Material

keaa027_Supplementary_Data
