# Supplementary material for: Predictors of presenteeism, absenteeism and job loss in patients commencing methotrexate or biologic therapy for rheumatoid arthritis
Source: Rheumatology (Oxford). 2020 Feb 25;59(10):2908–19. doi: 10.1093/rheumatology/keaa027 (PMC7516097; doi:10.1093/rheumatology/keaa027)
Supplement: keaa027_Supplementary_Data [file keaa027_supplementary_data.docx]

**SUPPLEMENTARY MATERIAL**

***Table 1 SOC codes of the patients in the analysis, stratified by cohort***

| **Work Category (SOC code, major group structure)** | **MTX-starters, N(%)** | **Biologic-starters, N(%)** |
| --- | --- | --- |
| 1. Managers, directors and senior officials | 38 (8.2) | 26 (10.0) |
| 2. Professional occupations | 73 (15.8) | 36 (13.9) |
| 3. Associated professional occupations and technical occupations | 65 (14.0) | 28 (10.8) |
| 4. Administrative and secretarial occupations | 61 (13.2) | 40 (15.4) |
| 5. Skilled trades occupations | 60 (13.0) | 21 (8.1) |
| 6. Caring, leisure and other service occupations | 52 (11.2) | 32 (12.3) |
| 7. Sales and customer service occupations | 40 (8.6) | 10 (3.9) |
| 8. Process, plant and machine operatives | 22 (4.8) | 4 (1.5) |
| 9. Elementary occupations | 36 (7.8) | 23 (8.9) |
| Uncoded | 16 (3.5) | 40 (15.4) |

*^a^Patients who did not provide enough information on their occupation to be coded using SOC*

*N: number; SOC: Standard Occupational Classification*

|  | No presenteeism, OR (95% CI) | | Presenteeism Score, IRR (95% CI) | |
| --- | --- | --- | --- | --- |
| Baseline Predictor | **MTX-starters**  **(N=427)** | **Biologic-starters**  **(N=239)** | **MTX-starters**  **(N=427)** | **Biologic-starters**  **(N=239)** |
| Age | 1.01 (0.98, 1.04) | 1.01 (0.99, 1.04) | 1.00 (0.99, 1.01) | 1.00 (0.99, 1.01) |
| Women vs men | 0.81 (0.46, 1.41) | 1.03 (0.55, 1.94) | 1.38 (1.13, 1.70) | 1.03 (0.80, 1.33) |
| Smoking  *Ex vs never*  *Current vs never* | 0.81 (0.45, 1.45)  0.55 (0.24, 1.24) | 1.36 (0.78, 2.37)  1.14 (0.51, 2.56) | 0.96 (0.78, 1.17)  0.98 (0.79, 1.22) | 1.09 (0.89, 1.33)  1.15 (0.86, 1.54) |
| SJC28 | 0.99 (0.94, 1.04) | 1.08 (1.02, 1.14) | 1.01 (0.99, 1.03) | 1.01 (0.99, 1.03) |
| HAQ | 0.43 (0.28, 0.67) | 0.48 (0.30, 0.77) | 1.36 (1.22, 1.53) | 1.47 (1.24, 1.75) |
| Pain VAS  *Natural scale*  *Standardised scale* | 0.98 (0.97, 0.99)  0.65 (0.48, 0.87) | 0.99 (0.98, 1.01)  0.84 (0.65, 1.15) | 1.01 (1.00, 1.01)  1.22 (1.11, 1.34) | 1.01 (1.00, 1.01)  1.14 (1.02, 1.27) |
| Fatigue VAS  *Natural scale*  *Standardised scale* | 0.98 (0.97, 0.98)  0.48 (0.37, 0.62) | 0.99 (0.98, 1.00)  0.81 (0.62, 1.07) | 1.01 (1.01, 1.01)  1.31 (1.19, 1.44) | 1.01 (1.00, 1.01)  1.23 (1.08, 1.40) |
| HADS Depression | 0.83 (0.76, 0.91) | 0.89 (0.83, 0.96) | 1.06 (1.04, 1.08) | 1.04 (1.01, 1.06) |
| HADS Anxiety | 0.86 (0.78, 0.95) | 0.89 (0.82, 0.96) | 1.04 (1.02, 1.06) | 1.03 (1.00, 1.05) |
| EQ5D (standardised) | 1.96 (1.22, 3.15) | 1.41 (1.04, 1.92) | 0.82 (0.75, 0.89) | 0.88 (0.81, 0.97) |
| NS-SEC  *Class 2 vs. Class 1*  *Class 3 vs. Class 1* | 1.25 (0.64, 2.46)  0.90 (0.47, 1.69) | 0.91 (0.41, 2.00)  1.23 (0.62, 2.43) | 0.87 (0.70, 1.08)  1.02 (0.84, 1.25) | 1.33 (1.03, 1.71)  1.29 (1.01, 1.65) |
| Comorbidity  *1 comorbidity vs 0*  *≥2 comorbidities vs 0*  *CI: confidence interval; DAS28: Disease Activity Score; HADS: Hospital Anxiety and Depression Scale; HAQ: Health Assessment Questionnaire; IRR: incidence rate ratio; NS-SEC: The National Statistics Socio-Economic Classification (see table 2 for definition of classes); OR: odds ratio; SJC: swollen joint count; VAS: visual analogue scale* | 0.84 (0.47, 1.50)  0.66 (0.29, 1.50) | 0.77 (0.39, 1.54)  0.44 (0.18, 1.07) | 1.06 (0.88, 1.28)  1.16 (0.90, 1.50) | 1.01 (0.81, 1.26)  1.14 (0.87, 1.49) |

***Table 2 Predictors of imputed presenteeism from univariable models***

|  | No presenteeism, OR (95% CI) | | Presenteeism Score, IRR (95% CI) | |
| --- | --- | --- | --- | --- |
| Baseline Predictor | **MTX-starters**  **(N=427)** | **Biologic-starters**  **(N=239)** | **MTX-starters**  **(N=427)** | **Biologic-starters**  **(N=239)** |
| Age | 1.01 (0.97, 1.05) | 1.02 (0.99, 1.06) | 1.00 (0.99, 1.01) | 1.00 (0.99, 1.01) |
| Women vs men | 1.12 (0.54, 2.30) | 1.49 (0.77, 2.89) | 1.24 (1.00, 1.53) | 0.97 (0.75, 1.25) |
| Smoking status vs never  *Former*  *Current* | 0.70 (0.35, 1.41)  0.47 (0.19, 1.18) | 1.60 (0.87, 2.97)  1.44 (0.57, 3.60) | 0.96 (0.79, 1.15)  1.02 (0.83, 1.25) | 1.05 (0.86, 1.29)  0.97 (0.71, 1.32) |
| HAQ | 0.66 (0.36, 1.21) | 0.44 (0.24, 0.82) | 1.09 (0.93, 1.27) | 1.32 (1.06, 1.64) |
| Pain-VAS  *Natural scale*  *Standardised scale* | 1.01 (0.99, 1.03)  1.30 (0.81, 2.09) | 1.01 (0.99, 1.03)  1.27 (0.86, 1.87) | 1.00 (0.997, 1.01)  1.05 (0.93, 1.18) | 1.00 (0.99, 1.01)  1.00 (0.88, 1.13) |
| Fatigue-VAS  *Natural scale*  *Standardised scale* | 0.98 (0.96, 0.99)  0.53 (0.33, 0.83) | 1.01 (0.99, 1.02)  1.15 (0.79, 1.69) | 1.01 (1.00, 1.01)  1.16 (1.00, 1.35) | 1.00 (0.999, 1.01)  1.12 (0.97, 1.30) |
| HADS General Distress | 0.96 (0.90, 1.02) | 0.94 (0.89, 0.99) | 1.01 (0.996, 1.02) | 1.00 (0.99, 1.02) |
| NS-SEC  *Class 2 vs. Class 1*  *Class 3 vs. Class 1* | 1.18 (0.53, 2.62)  0.90 (0.45, 1.80) | 1.07 (0.46, 2.47)  1.63 (0.76, 3.51) | 0.89 (0.73, 1.09)  1.05 (0.88, 1.26) | 1.23 (0.97, 1.56)  1.21 (0.95, 1.53) |
| Comorbidity  *1 comorbidity vs 0*  *≥2 comorbidities vs 0* | 0.93 (0.49, 1.78)  1.07 (0.40, 2.83) | 0.88 (0.41, 1.90)  0.46 (0.16, 1.29) | 1.03 (0.87, 1.23)  1.02 (0.80, 1.30) | 1.02 (0.91, 1.25)  0.98 (0.75, 1.29) |

*CI: confidence interval; DAS28: Disease Activity Score; HADS: Hospital Anxiety and Depression Scale; HAQ: Health Assessment Questionnaire; IRR: incidence rate ratio; NS-SEC: The National Statistics Socio-Economic Classification (see table 2 for definition of classes); OR: odds ratio; SJC: swollen joint count; VAS: visual analogue scale*

***Table 3 –Predictors of imputed presenteeism from multivariable models***

**RAMS and BRAGGSS coinvestigators**

BRAGGSS Co-investigators: Prof C Pitzalis (Barts Health NHS Trust, UK); Dr E Williams, Dr R K Moitra, Dr D J Shawe (Basingstoke & North Hampshire NHS Foundation Trust, UK); Dr. Wig (Bolton NHS Foundation Trust , UK); Dr R Laxminarayan (Burton Hospitals NHS Foundation Trust, UK); Prof I Bruce, Prof A Barton, Dr R Gorodkin, Dr P Ho, Prof K Hyrich, Dr F McKenna, Dr S Haque (Central Manchester University Hospitals NHS Foundation Trust, UK); Dr K Fairburn (Chesterfield Royal Hospital NHS Foundation Trust, UK); Dr J Nixon, Dr T Barnes, Dr M Hui (Countess of Chester Hospital NHS Foundation Trust, UK); Dr D Coady, Dr D Wright, Dr C Morley, Dr G Raftery, Dr C Bracewell (City Hospitals Sunderland NHS Foundation Trust, UK); Dr R Reece, Dr D. Armstrong, Dr A J Chuck, Dr S Hailwood, Dr N Kumar, Dr D Ashok (County Durham and Darlington NHS Foundation Trust, UK); Dr S C O'Reilly, Dr T Ding, Dr L J Badcock, Dr C M Deighton , Dr N Raj, Dr M R Regan, Dr G D Summers, Dr R A Williams (Derby Hospitals NHS Foundation Trust, UK); Dr C A Kelly, Dr J Hamilton, Dr C R Heycock, Dr V Saravanan, Mrs S Pugmire (Gateshead Health NHS Foundation Trust, UK); Dr M Green, Dr A Gough, Dr C Lawson (Harrogate & District NHS Foundation Trust, UK); Dr A Kuttikat, Dr D Parthajit, Dr E Borbas, Dr T Wazir (Kettering General Hospital NHS Foundation Trust, UK); Dr. Horton (Lancashire Care NHS Foundation Trust, UK); Prof P Emery, Dr S. Bingham, Prof A. Morgan, Prof H A Bird, Prof P G Conaghan, Dr C T Pease, Dr R J Wakefield, Prof M Buch, Dr S Dass (Leeds Teaching Hospitals NHS Trust, UK); Dr F N Birrell, Dr P R Crook (Northumbria Healthcare NHS Foundation Trust, UK); Dr B Szebenyi, Dr D Bates, Dr D James, Dr T Gillott, Dr A Alvi, C Grey, J Browning (Northern Lincolnshire and Goole Hospitals NHS Foundation Trust, UK); Dr J F McHale, Dr I C Gaywood, Dr A C Jones, Dr P Lanyon, Dr I Pande, Prof M Doherty, Dr A Gupta, Dr P A Courtney, Dr A Srikanth, Dr A Abhishek (Nottingham University Hospitals NHS Trust, UK); Dr L Das, Dr M Pattrick, Dr H N Snowden, Dr A P Bowden, Dr E E Smith, Dr P Klimiuk, Dr D J Speden (Pennine Acute Hospitals NHS Trust, UK); Dr J M Ledingham , Dr R G Hull, Dr F McCrae, Dr A Cooper, Dr S A Young Min, Dr Wong, Dr Shaban (Portsmouth Hospitals NHS Trust, UK); Prof A D Woolf, Dr M Davis, Dr D Hutchinson, Dr A Endean (Royal Cornwall Hospitals NHS Trust, UK); Dr D Mewar, Dr E J Tunn, Dr K Nelson, Dr T D Kennedy, Dr C Dubois (Royal Liverpool and Broadgreen University Hospitals NHS Trust); Dr J Pauling, Dr E Korendowych, Dr T Jenkinson, Dr R Sengupta, Dr A Bhalla, Prof N McHugh, Dr W Tillett, Dr T Ahmed (Royal National Hospital for Rheumatic Diseases NHS Foundation Trust, UK); Prof H Chinoy, Prof T O’Neil, Prof A Herrick, Prof A Jones, Dr R Cooper, Prof W Dixon, Dr B Harrison (Salford Royal NHS Foundation Trust, UK); Dr M Akil, Dr S Till, Dr L Dunkley, Dr R Tattersall, Dr R Kilding, Dr T Tait, Dr J Maxwell, Dr K-P Kuet (Sheffield Teaching Hospitals NHS Foundation Trust, UK); Dr M J Plant, Dr F Clarke, Dr J N Fordham, Dr S Tuck, Dr S K Pathare, Dr A Paul (South Tees Hospitals NHS Foundation Trust, UK); Dr C P Marguerie, Dr S P Rigby, Dr N Dunn (South Warwickshire General Hospital NHS Trust, UK); Dr S Hider, Dr A Menon, Dr C Dowson, Dr S Dutta, Dr S Kamath, Dr J Packham, Dr S Price, Dr E Roddy, Dr Z Paskins, Prof A. Hassell (Staffordshire & Stoke-on-Trent Partnership NHS Trust, UK); Dr A Ismail, Dr C Filer (Stockport NHS Foundation Trust, UK); Dr R Abernethy, Dr A R Clewes, Dr J K Dawson (St Helens and Knowsley Hospitals NHS Trust, UK); Prof G Kitas, Dr N Erb, Dr R Klocke, Dr A J Whallett, Dr K Douglas, Dr A Pace, Dr R Sandhu, Dr H John (The Dudley Group of Hospitals NHS Foundation Trust, UK); Dr S Lane (The Ipswich Hospital NHS Trust, UK); Prof J D Isaacs, Prof H Foster, Dr B Griffiths, Dr I Griffiths, Dr L Kay, Dr W-F Ng, Dr P N Platt, Dr D J Walker, Dr P Peterson, Dr A Lorenzi, Dr M Friswell, Dr B Thompson, Dr M Lee, Dr A Pratt (The Newcastle upon Tyne Hospitals NHS Foundation Trust, UK); Dr D Mulherin, Dr S V Chalam, , Dr T Price, Dr T Sheeran, Dr S Venkatachalam, Dr S Baskar, Dr S Raizada (The Royal Wolverhampton Hospitals NHS Trust, UK); Dr A Filer, Dr Bowman, Dr P Jobanputra Dr E C Rankin (University Hospital Birmingham NHS Foundation Trust, UK); Dr S Dubey, Dr K Chaudhuri, Dr A Price-Forbes, Dr J Ravindran (University Hospitals of Coventry and Warwickshire NHS Trust, UK); Dr A Moorthy, Dr P Sheldon, Dr W Hassan, Dr J Francis, Dr A Kinder, Dr R Neame (University Hospitals of Leicester NHS Trust, UK); Dr M Bukhari, Dr L Ottewell, Dr Palkonyai (University Hospitals of Morcambe Bay NHS Trust, UK); Dr D T O’Reilly, Dr V Rajagopal (West Suffolk Hospitals NHS Trust, UK); Dr E Gladston Chelliah (Wrightington, Wigan and Leigh Hospitals NHS Foundation Trust, UK); Dr M Green, Dr M Quinn, Dr A Isdale, Dr A Brown, Dr B Saleem, Dr Z Al-Saffar, Dr G Koduri (York Teaching Hospitals NHS Foundation Trust, UK),

RAMS co-investigators: Dr A Adebajo (Barnsley Hospital NHS Foundation Trust, UK); Dr F McKenna (Central Manchester University Hospitals NHS Foundation Trust, UK); Prof M Callan (Chelsea and Westminster Hospital NHS Foundation Trust, UK); Dr S Levy (Croydon Health Services NHS Trust, UK); Dr S Knight (East Cheshire NHS Trust, UK); Dr L-S Teh (East Lancashire Hospitals NHS Trust, UK); Dr J Hamilton, Dr V Saravanan (Gateshead Health NHS Foundation Trust, UK); Dr E Williams (Hampshire Hospitals NHS Foundation Trust, UK); Dr A Gough (Harrogate and District NHS Foundation Trust, UK); Dr J Galloway, Prof D Scott (King's College Hospital NHS Foundation Trust, UK); Dr L Macphie (Lancashire Care NHS Foundation Trust, UK); Dr L Pollard (Lewisham and Greenwich NHS Trust, UK); Prof D Symmons (Macclesfield District General Hospital, UK); Prof K Hyrich (Manchester University Hospitals NHS Foundation Trust, UK); Dr T Marshall (Norfolk and Norwich University Hospitals NHS Foundation Trust, UK); Dr M Perry, Dr L Robertson, Dr N Viner (Plymouth Hospitals NHS Trust, UK); Dr A Cooper (Portsmouth Hospitals NHS Trust, UK); Dr K Ahmed (Princess Alexandra Hospital NHS Trust, UK); Dr M Davis (Royal Cornwall Hospitals NHS Trust, UK); Prof H Chinoy, Prof D Symmons (Salford Royal NHS Foundation Trust, UK); Dr R Smith (Salisbury NHS Foundation Trust, UK); Dr C Mathews (South London Healthcare NHS Trust, UK); Dr C Marguerie (South Warwickshire NHS Foundation Trust, UK); Prof B Dasgupta (Southend University Hospital NHS Foundation Trust, UK); Dr S Hider, Dr S Kamath (Staffordshire and Stoke-on-Trent Partnership NHS Trust, UK); Dr D Roy (Tameside and Glossop Integrated Care NHS Foundation Trust, UK); Dr S Lane (The Ipswich Hospital NHS Trust, UK); Dr M Lee (The Newcastle upon Tyne Hospitals NHS Foundation Trust, UK); Dr S Naz (The Pennine Acute Hospitals NHS Trust, UK); Dr A Al-Ansari, Dr R Amarasena (The Robert Jones and Agnes Hunt Orthopaedic Hospital NHS Foundation Trust, UK); Dr G Smith (The Rotherham NHS Foundation Trust, UK); Dr B Quilty (The Royal Bournemouth and Christchurch Hospitals NHS Foundation Trust, UK); Dr N Viner (Torbay and South Devon NHS Foundation Trust, UK); Dr N Gullick (University Hospitals Coventry and Warwickshire NHS Trust, UK); Dr P Sanders (University Hospital of South Manchester NHS Foundation Trust, UK); Dr W Hassan (University Hospitals of Leicester NHS Trust, UK); Dr M Bukhari (University Hospitals of Morecambe Bay NHS Foundation Trust, UK); Dr E Gladston Chelliah (Wrightington, Wigan and Leigh NHS Foundation Trust, UK); Dr M Green (York Teaching Hospital NHS Foundation Trust, UK)
